# Illicit Drug Use and Smell and Taste Dysfunction: A National Health and Nutrition Examination Survey 2013–2014

**DOI:** 10.3390/healthcare10050909

**Published:** 2022-05-13

**Authors:** Hui-Han Kao, Hsi-Han Chen, Kuan-Wei Chiang, Sheng-Yin To, I-Hsun Li, Yu-Chieh Huang, Li-Ting Kao

**Affiliations:** 1 Graduate Institute of Life Sciences, National Defense Medical Center, Taipei 11490, Taiwan; hhkao@narlabs.org.tw; 2 Science and Technology Policy Research and Information Center, National Applied Research Laboratories, Taipei 10636, Taiwan; 3 School of Public Health, National Defense Medical Center, Taipei 11490, Taiwan; ein060113@gmail.com; 4 Department of Psychiatry, Yang Ji Mental Hospital, Keelung 20445, Taiwan; gracecute1213@gmail.com; 5 Department of Otolaryngology—Head and Neck Surgery, Taipei Veterans General Hospital, Taipei 11217, Taiwan; weiskyli@gmail.com; 6 Department of Pharmacy Practice, Tri-Service General Hospital, Taipei 11490, Taiwan; lhs01077@gmail.com; 7 School of Pharmacy, National Defense Medical Center, Taipei 11490, Taiwan; 8 Department of Pharmacology, National Defense Medical Center, Taipei 11490, Taiwan; 9 Department of Psychiatry, Tri-Service General Hospital, Taipei 11490, Taiwan

**Keywords:** illicit drug, cannabis, smell, taste, NHANES

## Abstract

Taste and smell dysfunction are suspected to be associated with substance use. However, representative epidemiological studies remain insufficient. This cross-sectional study explored the relationship between drug use (including cannabis or hashish, cocaine, heroin, and methamphetamine) and olfactory/gustatory dysfunction using data from the 2013–2014 National Health and Nutrition Examination Survey. In this study, participants who completed the smell examination with mean age of 59 were classified into four groups: cannabis users (*n* = 845), participants without cannabis use (*n* = 794), illicit drug users (*n* = 450), and participants without illicit drug use (*n* = 2000). Participants who completed the taste examination with mean age of 58 were also categorised into four groups: cannabis users (*n* = 810), participants without cannabis use (*n* = 714), illicit drug users (*n* = 428), and participants without illicit drug use (*n* = 1815). Logistic regression models investigated the association between cannabis or illicit drug use and smell or taste dysfunctions among study participants. Odds ratios and 95% confidence intervals were calculated. Finally, we did not find correlations between illicit drug use and dysfunction of taste or smell senses; our findings were consistent in many subgroup analyses. We recommend that further studies explore the mechanism and dose of illicit drug use that could have chemosensory impacts.

## 1. Introduction

Increasing evidence indicates that illicit drug use may lead to a substantial loss of life and disabilities [1]. Up until 2019, it was estimated that 5.4% of the global population used illegal drugs, and around 36.3 million people worldwide could be considered problematic drug users [2,3]. Additionally, according to the United Nations Office on Drugs and Crime report, cannabis is the most used drug, followed by opioids and amphetamines [2]. Moreover, drug abuse has noted tremendous impacts and health problems, including socioeconomic burdens, social and legal consequences, poverty and mental disorders, etc. [4,5]. Previous research indicated the need for excessive sensory stimulation as a predisposition to addictive behaviors. Exploring more sensory characteristics in people with addictive behaviors might provide the governments and policymakers with more information on this population [1,6,7].

Generally, olfactory dysfunction has been recognised as a heterogeneous condition, commonly caused by infection and trauma [8]. Olfactory loss is divided into two types: conductive and sensorineural dysfunction [9]. Impaired olfaction is disabling because it results in nutritional alterations in patients, increases the risk of injury, and lowers social relationships with reduced quality of life [10,11]. To date, taste and smell dysfunction are suspected to be associated with substance use. However, representative epidemiological studies remain insufficient. Smell and taste dysfunctions involve ageusia, hypogeusia, anosmia, hyposmia, etc. Ageusia refers to an absence of the sense of taste and hypogeusia refers to decreased taste sensitivity [12]. Furthermore, qualitative gustatory dysfunction is more frequent than quantitative dysfunction [13]. Moreover, anosmia and hyposmia, referring to a total and partial loss of the senses of smell, affect up to 25% of adults who are 50 years or older in America [14,15,16,17].

Currently, most studies explored the association of smell and taste dysfunction in substance users with alcohol use and smoking [18,19,20,21]. Some studies have shown a preserved level of olfaction without odour detection thresholds or discrimination deficits in alcoholic participants. Still, some even revealed impaired olfactory event-related potentials and olfactory dysfunction in patients with alcohol dependence [22,23]. However, only a few case series and animal models have reported and investigated the effects of chemosensory impairment in cannabis use [24,25]. Some studies also noticed that heroin addicts might be associated with a change in smell and taste function [26,27]. Nevertheless, there were equivocal results in reporting the connection between cocaine use and smell/taste dysfunction from animal models and humans [28,29].

Accordingly, relevant findings in previous studies have remained inconsistent. The research design investigating the association between chemosensory dysfunction and substance use has included cell research, animal models, and case-control studies. However, although the research focused on one illicit drug, the collected data were limited by small sample sizes and patient reluctance to discuss illicit drug use or resulting harms. Moreover, comprehensive epidemiological studies investigating the relationship between illicit drug use and smell and taste dysfunction have remained deficient. Therefore, this study explored the relationship between drug use (including cannabis or hashish, cocaine, heroin, and methamphetamine) and olfactory/gustatory dysfunction using information from a large-scale cross-sectional database in the United States.

## 2. Materials and Methods

### 2.1. Database

Data from the National Health and Nutrition Examination Survey (NHANES) in the United States were used in this cross-sectional study. NHANES, a vital programme of the National Center for Health Statistics (NCHS), contains interviews and physical examination data of health and nutrition measurements. The database also includes information on about 5000 people from counties across the U.S. as a national and yearly representative sample. The research protocols for the NHANES received approval from the NCHS Research Ethics Review Board and written informed consent was obtained from each participant (https://www.cdc.gov/nchs/nhanes/irba98.htm, accessed 1 April 2022). This study was exempted from a full review of the Institutional Review Board because the data used were de-identified secondary data from NHANES.

### 2.2. Study Sample Selection

**Figure 1:**
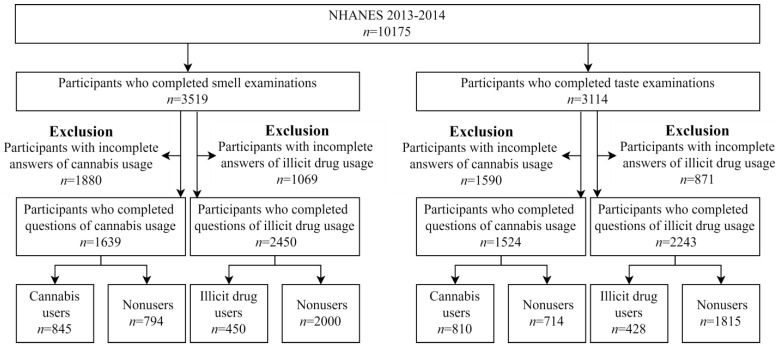
Flow Diagram for Study Sample Selection.

This study investigated illicit drugs and cannabis use associated with smell and taste dysfunction. Therefore, participants who underwent the Taste and Smell Examination and filled out the Drug Use Questionnaire from 2013 to 2014 were included. Those with incomplete smell and taste, as detected from the examination results, or those with incomplete responses to related drug use questions, were excluded from this study. Finally, participants who completed the smell examination were classified into four groups: cannabis users (*n* = 845), participants without cannabis use (*n* = 794), illicit drug users (*n* = 450), and participants without illicit drug use (*n* = 2000). Participants who completed the taste examination were also categorised into four groups: cannabis users (*n* = 810), participants without cannabis use (*n* = 714), illicit drug users (*n* = 428), and participants without illicit drug use (*n* = 1815). Cannabis and illegal drug users were identified based on their answers to the relevant questions in the Drug Use Questionnaire. Therefore, cannabis users were defined as participants who answered that they had ever used marijuana or hashish, and illicit drug users were identified as participants who responded that they had ever used cocaine, heroin, or methamphetamine. Flow diagram for study sample selection is displayed in Figure 1.

### 2.3. Outcome Measurements

The definitions of smell and taste dysfunction were based on the smell and taste examination results. An odour identification test examined the ability to smell, and the tasting ability was measured using salt and quinine taste testing.

#### 2.3.1. Smell Dysfunction Definition

An eight-item odour identification test was used to identify the participants’ ability to smell. First, participants were presented with eight specific odours via scratching of test strips. Then, eight specific scents were presented in a fixed sequence of chocolate, strawberry, smoke, leather, soap, grape, onion and natural gas. Participants were finally requested to choose the correct odour of the four listed options for each scent. Those who failed to identify six or more odours were defined as participants with smell dysfunction.

#### 2.3.2. Taste Dysfunction Definition

The 1-mM quinine whole-mouth taste test was used to determine participants’ taste ability [30]. The participants were first asked to take the 10-mL quinine solution into their mouths without swallowing, after which they were asked to swish and spit out the solution. Subsequently, each participant identified the taste of the solution, and their mouths were rinsed with water afterward. Those who failed to identify the bitter taste of quinine in the 1-mM quinine whole-mouth taste test were defined as participants with taste dysfunctions.

### 2.4. Covariate Measurement

We reduced the influence of potential confounders by considering participants’ age, gender, ethnicity, hypertension status, diabetes mellitus status, coronary heart disease history, angina pectoris, heart attack susceptibility, stroke, persistent cold/flu over the last 12 months before the study, head injury/loss of consciousness, broken nose/serious injuries to the face or skull, two or more sinus infections, smoking status, heavy alcohol use, and overweight in the regression models. Ethnicity was categorised as Mexican American, other Hispanic, non-Hispanic white, non-Hispanic black, non-Hispanic Asian and other races. Participants were defined as being overweight or having a medical history of hypertension, diabetes mellitus, coronary heart disease, angina pectoris, heart attack and stroke, based on their self-report of doctors’ diagnosis. Those taking insulin and diabetic pills were also defined as having diabetes mellitus. Furthermore, participants who smoke and heavily take alcohol, or those with persistent cold/flu over the last 12 months before the study, head injury/loss of consciousness, broken nose/serious injury to the face or skull and those with two or more sinus infections, were identified using self-reported questionnaires.

### 2.5. Statistical Analysis

This study used the SAS system (SAS System for Windows, V.9.4, SAS Institute Inc., Cary, NC, USA) to conduct all analyses. Chi-squared tests were first applied to investigate differences in gender, ethnicity, hypertension, diabetes mellitus, coronary heart disease, angina pectoris, heart attack, stroke, persistent cold/flu last 12 months before the study, head injury/loss of consciousness, broken nose/serious injury to face or skull, two or more sinus infections, smoking status, heavy alcohol use, and overweight, between participants with and without smell or taste dysfunction. Next, the independent t-test was performed to compare the difference in age between participants with and without smell or taste dysfunctions. Finally, logistic regression models investigated the association between cannabis or illicit drug use and smell or taste dysfunctions among study participants, after which odds ratios (ORs) and 95% confidence intervals (CIs) were calculated. A two-sided *p*-value < 0.05 was used to define the statistical significance of this study.

## 3. Results

This study contained smell and taste dysfunction study groups. Table 1 displays study participants’ demographic characteristics and comorbidities with and without smell dysfunctions. The relevant findings showed significant differences in age, gender, ethnicity, hypertension, diabetes mellitus, coronary heart disease, angina pectoris, heart attack, stroke, two or more sinus infections, smoking status, and overweight between participants with and without smell dysfunction. The results (Table 1) also showed significant differences between the participants with and without taste dysfunction based on age, ethnicity, and smoking status. Therefore, we considered all these factors in the regression model to eliminate potential bias.

Table 2 presents the prevalence of cannabis and illicit drug use among participants with and without smell dysfunction to further investigate the cannabis and illicit drug use association with smell dysfunction. Participants with and without smell dysfunction comprised 40.8% and 52.7% of cannabis users, respectively. After adjustments, the adjusted OR for cannabis use was 0.67 (95% CI, 0.43–1.03) among participants with and without smell dysfunction. Furthermore, individuals with and without smell dysfunction included 15.6% and 18.8% of illicit drug users, respectively. However, the adjusted OR for illicit drug use was 0.85 (95% CI, 0.59–1.25) among study participants with and without smell dysfunction, respectively. Subsequently, we further estimated the adjusted ORs for cocaine (OR, 0.85; 95% CI, 0.58–1.26), heroin (OR, 0.69; 95% CI, 0.30–1.56), and methamphetamine (OR, 0.84; 95% CI, 0.47–1.50) use of the understudied participants, respectively. Overall, there was no significant difference in smell dysfunction between cannabis or illicit drug users and those who did not use cannabis or illicit drugs.

The prevalence and ORs for cannabis and illicit drug use among study participants with and without taste dysfunction are displayed in Table 2. Study participants with and without taste dysfunction comprised 57.0% and 52.2% of cannabis users, respectively. However, the adjusted OR for cannabis use was 0.98 (95% CI, 0.71–1.35) among participants with and without taste dysfunction. Furthermore, although participants with and without taste dysfunction comprised 20.5% and 18.8% of illicit drug users, respectively, the adjusted OR for illicit drug use was 0.85 (95% CI, 0.63–1.15) among participants with and without taste dysfunction. We further evaluated the adjusted ORs for cocaine (OR, 0.92; 95% CI, 0.68–1.26), heroin (OR, 0.64; 95% CI, 0.32–1.27), and methamphetamine (OR, 0.79; 95% CI, 0.50–1.25) use of the participants. Summarily, no significant difference in taste dysfunction was observed between cannabis or illicit drug users and participants who did not use cannabis or illicit drugs.

Subsequently, to reduce the effect of gender, we performed stratified analyses to explore the relationship between cannabis or illicit drug use and smell or taste dysfunction in males and females (Table 3). After adjusting for various confounders, findings indicated no significant association between cannabis or illicit drug use and smell or taste dysfunctions in the male or female population. Then, due to the influence of age on smell and taste dysfunction, we further investigated the association between cannabis or illicit drug use and smell or taste dysfunctions among study participants according to the different age groups (Table 4). The study results remained unchanged after adjusting for demographic characteristics and comorbidities. Additionally, there was no significant difference in smell or taste dysfunction between cannabis or illicit drug users and those who did not use cannabis or illicit drugs in participants aged 40–49, 50–59, and 60–69 years.

Furthermore, to consider the effect of the duration of drug use and the age of first drug use, we also investigated the association between cannabis/illicit drug use and smell/taste dysfunction among study participants after adjusting for age of first cannabis/illicit drug use, days used cannabis/illicit drug during the past 30 days, and other demographic characteristics and comorbidities (Supplemental Table S1). The findings remained unchanged after adjusting for relevant confounders.

## 4. Discussion

To date, many medications, such as antimicrobials, antihypertensives, antidepressants, antipsychotics, antineoplastics, agents in cigarette smoke, and ethanol use, have been associated with chemosensory disturbances [31,32]. The potential olfactory dysfunction mechanisms based on these substances may be due to olfactory epithelium damage or central nervous system damage. Based on clinical experiences, this study initially hypothesised that illicit drug use might be associated with smell and taste function changes. However, we observed no association between participants with smell or taste dysfunction and cannabis or illicit drug use history. Notably, the association did not remain statistically significant after adjustments for confounders, including age, race, gender, or other physical comorbidities in males, females, and participants with different age groups. 

Previous studies have investigated the relationship between substance use and smell/taste function. However, most current research only examined the impact of smoking and alcohol on taste or smell functions. Therefore, potential impacts on olfactory function have remained controversial. For instance, a study enrolled 48 drug addicts between the ages of 16 and 48. Although 52.1% of the participants showed disturbances in olfactory performance, 16.7% were diagnosed with ageusia. Additionally, olfactory problems were detected in those who took drugs intravenously and smoked or inhaled drugs [33]. Another study enrolled 21 smokers and 59 non-smokers. Although their results showed smaller olfactory bulb volumes in smokers than in non-smokers, no difference in olfactory function between the two groups was observed [18]. Moreover, though Schriever et al. suggested that smoking negatively affects the olfactory system before it becomes obvious in decreased olfactory function, Vennemann et al. noted that heavy smokers (20 or more cigarettes per day) had an increased risk for impairments in smell and taste senses [19]. Therefore, since the temporal lobe mediates olfactory processing, and a correlation between general olfactory function and olfactory bulb volumes has been proposed, factors that affect the optimal function of this system could result in issues [9].

Our study focused on the relationship between cannabis, illicit drug use, and smell or taste function changes. Some prior studies have also investigated the relevant issue of cannabis. As reported, cannabinoids are involved in the neuromodulatory regulation of the sensory systems [34,35]. The active ingredient of cannabis, delta-9-tetrahydrocannabinol (THC), has also been reported to palliate symptoms in cancer patients [36,37,38]. Furthermore, it stimulates the orosensory reward pathway and enhances food enjoyment. Although cannabinoid type-1 receptors are located in different brain olfactory areas, including the olfactory epithelium and bulb [39,40], cannabinoid receptor agonists increase the hedonic reactions to sweet taste and reduce the aversive responses to quinine [24,35,36,41]. However, participants in that research were cancer patients who were explicitly administered THC. Case reports from students with an experience of marijuana intoxication also reported more vivid taste sensations and a richer sense of smell when intoxicated [42]. Similarly, a recent study showed that cannabidiol (CBD) protects against the psychoactive effects of THC [43]. Moreover, Woelfl et al. conducted a randomised controlled trial, which showed that CBD intake did not affect healthy volunteers, and a single dose of CBD before taking THC administration was insufficient to mitigate the impact of THC [44]. Nevertheless, cannabis contains over 400 chemical entities, with the ratios of each compound in recreational cannabis making it challenging to investigate the effect of drug users in real-world situations [45,46]. Our study did not notice a change in the smell or taste of individuals who ever used cannabis. 

Additionally, our study did not find correlations between illicit drug use and dysfunction of taste or smell. Some previous research also investigated relevant issues. For instance, cocaine is usually administered illegally from the nasal route, and abusers often complain of decreased olfaction. Clinical pathology that contributes to reduced olfaction includes immune-mediated diseases and even nasal defects that require surgical reconstruction [47,48]. However, no olfactory or gustatory function tests were conducted on those users. Roebber et al. suggested that cocaine exposure did not change the taste sensitivity of animal models of mice [28]. Another study by Gordon et al. reported from three olfaction tests that most cocaine abusers did not develop permanent olfactory dysfunction [49]. Beidler and Smallman also proposed that nerves innervating the taste buds could regenerate after abrasion, making the taste buds retain function [50]. Therefore, there might be a restoration of chemosensory perception after quitting illicit drug use. Nevertheless, exploring the underlying mechanisms of individuals after using illicit drugs is needed. 

Furthermore, Perl et al. noticed that heroin addicts estimated sweet tastes and savoury smells as more pleasant, and bitter, sour or putrid tastes and odours were considered less unpleasant by them than detoxified former addicts and healthy controls [26]. A randomised controlled trial with a small sample size measured sweet and salt taste perceptions of heroin users, recently detoxified subjects, and healthy volunteers. The results showed that heroin users and recently detoxified subjects had significantly greater measures of taste perception, and even this effect could be reversed by opiate antagonists [27]. These results suggest that heroin addicts might have altered taste and odour hedonic brain mechanisms. Findings in previous literature were similar to the trends found in our study. We observed that individuals with and without smell dysfunction included 2.4% and 3.2% of heroin users, respectively. Even though the relationship did not reach statistical significance, heroin users were more sensitive to odours. With amphetamine, a scarce study investigated the association between smell or taste dysfunction and amphetamine use. 

This study had some unique strengths. First, it included a large-scale sample size from a population with a history of illicit drug use and relatively accurate examination results on smell and taste function. Second, we used both self-reported questionnaires and examinations (including an 8-item odour identification test and 1-mM quinine whole-mouth taste identification test) to identify the smell and taste dysfunctions, eliminating recall bias from respondents. Finally, this study investigated the relationships between smell/taste dysfunction and illicit drugs, including cannabis or hashish, cocaine, heroin, and methamphetamine. Moreover, based on our extensive literature search, this research is the first to comprehensively study the relationship between different illicit drugs and smell/taste dysfunction. Notwithstanding, several limitations were encountered in this study. First, we could not explore the causal relationships because of the cross-sectional nature of this study. Second, this study did not consider certain medications that may affect smell and taste because the database lacked detailed information. 

## 5. Conclusions

In conclusion, our research provided insight into the relationship between illicit drug use and olfactory/gustatory functions, and this association did not remain statistically significant after adjustments for confounders, including age, race, gender, or other physical comorbidities in males, females and participants with different age groups. With the diversity and complexity of chemical compounds in each illicit drug, studies with multidisciplinary teamwork may be warranted to objectively assess the nature of chemosensory effects caused by each illicit drugs. Therefore, we recommend that future studies explore the mechanism and dose of illicit drug use that could have chemosensory impacts in humans.

## Figures and Tables

**Table 1:** Baseline characteristics based on smell and taste dysfunction among study participants.

Variable	Participants with Smell Dysfunction (*n* = 630)		Participants without Smell Dysfunction (*n* = 2889)		*p* Value	Participants with Taste Dysfunction (*n* = 540)		Participants without Taste Dysfunction (*n* = 2574)		*p* Value
	No.	%	No.	%		No.	%	No.	%	
Age (Mean ± SD)	65.9 ± 12.2		57.5 ± 11.5		<0.001	57.0 ± 11.7		58.8 ± 12.0		0.002
Gender					<0.001					0.787
Male	368	58.4	1311	45.4		259	48.0	1251	48.6	
Female	262	41.6	1578	54.6		281	52.0	1323	51.4	
Ethnicity					0.030					<0.001
Mexican American	80	12.7	380	13.2		60	11.1	336	13.1	
Other Hispanic	61	9.7	249	8.6		46	8.5	214	8.3	
Non-Hispanic White	248	39.4	1316	45.6		253	46.9	1187	46.1	
Non-Hispanic Black	153	24.3	574	19.9		138	25.6	494	19.2	
Non-Hispanic Asian	78	12.4	307	10.6		26	4.8	297	11.5	
Other Race—Including Multi-Racial	10	1.6	63	2.2		17	3.2	46	1.8	
Ever had diagnosis										
Hypertension	365	57.9	1365	47.3	<0.001	266	49.4	1240	48.2	0.625
Diabetes mellitus	146	23.2	490	17.0	<0.001	85	15.8	444	17.3	0.406
Coronary heart disease	50	8.0	157	5.5	0.013	35	6.5	146	5.7	0.468
Angina pectoris	33	5.3	89	3.1	0.007	14	2.6	97	3.8	0.182
Heart attack	53	8.4	151	5.2	0.002	35	6.5	137	5.3	0.280
Stroke	65	10.3	109	3.8	<0.001	24	4.5	112	4.4	0.922
Persistent cold/flu last 12 months	48	7.6	188	6.5	0.314	32	5.9	173	6.7	0.503
Head Injury/Loss of consciousness	86	13.7	404	14.0	0.823	81	15.0	364	14.2	0.619
Broke Nose/Serious Injury to Face/Skull	91	14.5	412	14.3	0.875	87	16.1	371	14.4	0.318
Two or more sinus infections	155	24.8	1040	36.1	<0.001	185	34.6	903	35.1	0.811
Smoking status ^a^					0.039					0.004
Current smoker	98	15.6	543	18.8		125	23.2	442	17.2	
Former smoker	201	32.0	798	27.6		153	28.3	744	28.9	
Never	330	52.5	1548	53.6		262	48.5	1387	53.9	
Heavy alcohol use	95	21.7	422	18.1	0.079	86	19.1	389	18.7	0.864
Overweight	214	34.0	1160	40.2	0.004	204	37.8	1015	39.4	0.474

Note: SD, standard deviation. ^a^ The sum of *n* did not equal to the total number because of the missing data.

**Table 2:** Prevalence and odds ratios for cannabis and illicit drug use among study participants with and without smell and taste dysfunction.

Variables		Participants with Smell Dysfunction		Participants without Smell Dysfunction		Participants with Taste Dysfunction		Participants without Taste Dysfunction	
		No.	%	No.	%	No.	%	No.	%
Ever used cannabis or hashish									
	Yes	64	40.8	781	52.7	171	57.0	639	52.2
	No	93	59.2	701	47.3	129	43.0	585	47.8
	Crude OR (95% CI) ^a^	0.62 ** (0.44–0.86)				1.21 (0.94–1.57)			
	Adjusted OR (95% CI) ^a,b^	0.67 (0.43–1.03)				0.98 (0.71–1.35)			
Ever used illicit drug ^c^									
	Yes	46	15.6	404	18.8	83	20.5	345	18.8
	No	249	84.4	1751	81.3	322	79.5	1493	81.2
	Crude OR (95% CI) ^a^	0.80 (0.57–1.12)				1.12 (0.85–1.46)			
	Adjusted OR (95% CI) ^a,b^	0.85 (0.59–1.25)				0.85 (0.63–1.15)			
Type of illicit drug use									
Ever used cocaine									
	Yes	42	14.2	376	17.5	81	20.0	317	17.3
	No	253	85.8	1778	82.5	324	80.0	1520	82.7
	Crude OR (95% CI) ^a^	0.79 (0.56–1.11)				1.20 (0.91–1.57)			
	Adjusted OR (95% CI) ^a,b^	0.85 (0.58–1.26)				0.92 (0.68–1.26)			
Ever used heroin									
	Yes	7	2.4	69	3.2	11	2.7	62	3.4
	No	288	97.6	2084	96.8	394	97.3	1775	96.6
	Crude OR (95% CI) ^a^	0.73 (0.33–1.61)				0.80 (0.42–1.53)			
	Adjusted OR (95% CI) ^a,b^	0.69(0.30–1.56)				0.64(0.32–1.27)			
Ever used methamphetamine									
	Yes	16	5.4	154	7.2	30	7.4	137	7.5
	No	279	94.6	2001	92.9	375	92.6	1701	92.6
	Crude OR (95% CI) ^a^	0.75 (0.44–1.27)				0.99 (0.66–1.50)			
	Adjusted OR (95% CI) ^a,b^	0.84 (0.47–1.50)				0.79 (0.50–1.25)			

Note: CI = confidence interval; OR = odds ratio; ^a^ Logistic regression; ^b^ adjusted for age, gender, ethnicity, hypertension, diabetes mellitus, coronary heart disease, angina pectoris, heart attack, stroke, persistent cold/flu last 12 months, head injury/loss of consciousness, broke nose/serious injury to face/skull, two or more sinus infections, smoking status, heavy alcohol use, and overweight; ^c^ Study participants who ever used cocaine/heroin/methamphetamine. ** *p* ≤ 0.01

**Table 3:** Association between cannabis/illicit drug use and smell/taste dysfunction among study participants according to gender.

Variables		Participants with Smell Dysfunction		Participants without Smell Dysfunction		Participants with Taste Dysfunction		Participants without Taste Dysfunction	
		No.	%	No.	%	No.	%	No.	%
Male									
Ever used cannabis or hashish									
	Yes	43	47.3	395	57.3	88	61.5	336	56.6
	No	48	52.8	295	42.8	55	38.5	258	43.4
	Crude OR (95% CI) ^a^	0.67 (0.43–1.04)				1.23 (0.85–1.79)			
	Adjusted OR (95% CI) ^a,b^	0.76 (0.44–1.34)				1.21 (0.77–1.92)			
Ever used illicit drug ^c^									
	Yes	35	20.7	228	22.9	47	24.7	204	22.9
	No	134	79.3	770	77.2	143	75.3	686	77.1
	Crude OR (95% CI) ^a^	0.88 (0.59–1.32)				1.11 (0.77–1.59)			
	Adjusted OR (95% CI) ^a,b^	0.98 (0.62–1.54)				0.90 (0.60–1.35)			
Female									
Ever used cannabis or hashish									
	Yes	21	31.8	386	48.7	83	52.9	303	48.1
	No	45	68.2	406	51.3	74	47.1	327	51.9
	Crude OR (95% CI) ^a^	0.49 ** (0.29–0.84)				1.21 (0.85–1.72)			
	Adjusted OR (95% CI) ^a,b^	0.58 (0.28–1.20)				0.74 (0.46–1.19)			
Ever used illicit drug ^c^									
	Yes	11	8.7	176	15.2	36	16.7	141	14.9
	No	115	91.3	981	84.8	179	83.3	807	85.1
	Crude OR (95% CI) ^a^	0.53 (0.28–1.01)				1.15 (0.77–1.72)			
	Adjusted OR (95% CI) ^a,b^	0.69 (0.33–1.45)				0.83 (0.52–1.33)			

Note: CI = confidence interval; OR = odds ratio; ^a^ Logistic regression; ^b^ adjusted for age, ethnicity, hypertension, diabetes mellitus, coronary heart disease, angina pectoris, heart attack, stroke, persistent cold/flu last 12 months, head injury/loss of consciousness, broke nose/serious injury to face/skull, two or more sinus infections, smoking status, heavy alcohol use, and overweight; ^c^ Study participants who ever used cocaine/heroin/methamphetamine. ** *p* ≤ 0.01.

**Table 4:** Association between cannabis/illicit drug use and smell/taste dysfunction among study participants according to age.

Variables		Participants with Smell Dysfunction		Participants without Smell Dysfunction		Participants with Taste Dysfunction		Participants without Taste Dysfunction	
		No.	%	No.	%	No.	%	No.	%
40–49 years									
Ever used cannabis or hashish									
	Yes	19	29.7	378	49.2	86	53.4	295	47.5
	No	45	70.3	391	50.9	75	46.6	326	52.5
	Crude OR (95% CI) ^a^	0.44 ** (0.25–0.76)				1.27 (0.90–1.79)			
	Adjusted OR (95% CI) ^a,b^	0.66 (0.32–1.35)				0.97 (0.63–1.52)			
Ever used illicit drug ^c^									
	Yes	7	10.9	145	18.9	33	20.5	115	18.5
	No	57	89.1	624	81.1	128	79.5	506	81.5
	Crude OR (95% CI) ^a^	0.53 (0.24–1.18)				1.13 (0.74–1.75)			
	Adjusted OR (95% CI) ^a,b^	0.72 (0.29–1.80)				0.94 (0.57–1.55)			
50–59 years									
Ever used cannabis or hashish									
	Yes	45	48.4	403	56.5	85	61.2	344	57.1
	No	48	51.6	310	43.5	54	38.9	259	43.0
	Crude OR (95% CI) ^a^	0.72 (0.49–1.11)				1.19 (0.81–1.73)			
	Adjusted OR (95% CI) ^a,b^	0.69 (0.37–1.20)				1.04 (0.64–1.68)			
Ever used illicit drug ^c^									
	Yes	22	23.9	163	22.8	36	25.7	141	23.3
	No	70	76.1	552	77.2	104	74.3	463	76.7
	Crude OR (95% CI) ^a^	1.06 (0.64–1.77)				1.14 (0.74–1.74)			
	Adjusted OR (95% CI) ^a,b^	1.07 (0.59–1.97)				0.89 (0.54–1.47)			
60–69 years									
Ever used illicit drug ^c^									
	Yes	17	12.2	96	14.3	14	13.5	89	14.5
	No	122	87.8	575	85.7	90	86.5	524	85.5
	Crude OR (95% CI) ^a^	0.84 (0.48–1.45)				0.92 (0.50–1.68)			
	Adjusted OR (95% CI) ^a,b^	0.74 (0.40–1.39)				0.68 (0.34–1.35)			

Note: CI = confidence interval; OR = odds ratio; ^a^ Logistic regression; ^b^ adjusted for gender, ethnicity, hypertension, diabetes mellitus, coronary heart disease, angina pectoris, heart attack, stroke, persistent cold/flu last 12 months, head injury/loss of consciousness, broke nose/serious injury to face/skull, two or more sinus infections, smoking status, heavy alcohol use, and overweight; ^c^ Study participants who ever used cocaine/heroin/methamphetamine. ** *p* ≤ 0.01.

## Data Availability

Publicly available datasets were analyzed in this study. This data can be found here: https://wwwn.cdc.gov/nchs/nhanes/Default.aspx, accessed 1 April 2022.

## Supplementary Materials

The following supporting information can be downloaded at: https://www.mdpi.com/article/10.3390/healthcare10050909/s1, Table S1. Association between cannabis/illicit drug use and smell/taste dysfunction among study participants after adjusting for age of first cannabis/ illicit drug use, days used cannabis/ illicit drug during the past 30 days and other demographic characteristics and comorbidities.
