# Supplementary material for: Illicit Drug Use and Smell and Taste Dysfunction: A National Health and Nutrition Examination Survey 2013–2014
Source: Healthcare (Basel). 2022 May 13;10(5):909. doi: 10.3390/healthcare10050909 (PMC9140567; doi:10.3390/healthcare10050909)
Supplement: Supplementary file 1 [file healthcare-10-00909-s001.zip › healthcare-1718450-supplementary.pdf]

Supplemental Table S1. Association between cannabis/ illicit drug use and smell/taste dysfunction among study participants after adjusting for age of first cannabis/ illicit drug use, days used cannabis/ illicit drug during the past 30 days and other demographic characteristics and comorbidities

Variables	Participants with Smell Dysfunction	Participants without Smell Dysfunction	Participants with Taste Dysfunction	Participants without Taste Dysfunction
	Adjusted OR (95% CI) [a, b]		Adjusted OR (95% CI) [a, b]	
Ever used cannabis or hashish	0.61 (0.17-2.26)		1.67 (0.73-3.81)	
Type of illicit drug use				
Ever used cocaine	1.39 (0.27-7.09)		2.71 (0.66-11.23)	
Ever used heroin	- [c]		- [c]	
Ever used methamphetamine	0.33 (0.01-9.40)		2.55 (0.53-12.19)	

Note: CI= confidence interval; OR=odds ratio; [a] Logistic regression [b] Adjusted for age of first cannabis/ illicit drug use, days used cannabis/ illicit drug during the past 30 days, age, gender, ethnicity, hypertension, diabetes mellitus, coronary heart disease, angina pectoris, heart attack, stroke, persistent cold/flu last 12 months, head injury/loss of consciousness, broke nose/serious injury to face/skull, two or more sinus infections, smoking status, heavy alcohol use, and overweight. [c] - Represented the odds ratios could not be estimated because of the missing data. * $P \leq 0.05$, ** $P \leq 0.01$, *** $P \leq 0.001$.
